# First-Line Treatment with a Cyclin-Dependent Kinase 4/6 Inhibitor Plus an Aromatase Inhibitor for Metastatic Breast Cancer in Alberta

**DOI:** 10.3390/curroncol28030209

**Published:** 2021-06-18

**Authors:** Carla P. Amaro, Atul Batra, Sasha Lupichuk

**Affiliations:** 1 Tom Baker Cancer Centre, Calgary, AB T2N 1N4, Canada; carla.piresamaro@ahs.ca; 2 All India Institute of Medical Sciences, New Delhi 110029, India; batraatul85@gmail.com

**Keywords:** metastatic breast cancer, palbociclib, ribociclib, real-world, Canada, median time to progression on second-line treatment

## Abstract

In this analysis, we describe population-based outcomes for first-line treatment with a cyclin-dependent kinase 4/6 inhibitor (CDK4/6i) combined with an aromatase inhibitor (AI). All patients who were prescribed CDK4/6i + AI from January 2016 through June 2019 were included. Patient demographics, tumour and treatment characteristics were collected and described. Survival distributions were estimated using the Kaplan–Meier method. Multivariate analysis (MVA) was constructed to examine associations between potentially prognostic clinical variables and progression-free survival (PFS). In total, 316 patients were included. The median age was 61 years. After a median follow-up of 28.1 months, the median PFS was 37.9 months (95% CI, 26.7–NR). In the MVA, PR-negative tumour (HR, 2.37; 95% CI, 1.45–3.88; *p* = 0.001) and CDK4/6i dose reduction (HR, 1.51; 95% CI, 1.06–2.16; *p* = 0.022) predicted worse PFS. Median overall survival (OS) was not reached. The 30-month and 36-month OS rates were 74% and 68%, respectively. Of patients who progressed, 89% received second-line treatment. Median time to progression on second-line chemotherapy was 9.0 (5.8–17.6) months, and median time to progression on second-line hormonal therapy +/− targeted agent was 4.0 (3.4–8.6) months (*p* = 0.012). CDK4/6i + AI as first-line treatment for HR-positive, HER2-negative MBC in Alberta is justified based on favourable PFS and early OS outcomes.

## 1. Introduction

Breast cancer is the most commonly diagnosed malignancy and a major cause of death among women worldwide [1]. In Canada, approximately 27,400 women were diagnosed and 5100 died of breast cancer in 2020 [2]. Hormone receptor (HR)-positive and human epidermal growth factor receptor-2 (HER2)-negative breast cancer is the most common subtype, accounting for 60–70% of all cases [3].

The treatment of HR-positive, HER2-negative metastatic breast cancer (MBC) has undergone a paradigm shift in recent years. The combination of a cyclin-dependent kinase 4/6 inhibitor (CDK4/6i) with an aromatase inhibitor (AI) has emerged as the standard first-line treatment in patients with de novo stage IV disease and in those with recurrent metastatic disease who completed adjuvant antiestrogen therapy with an AI at least 12 months prior. Palbociclib was the first CDK4/6 inhibitor (CDK4/6i) to be approved in combination with an AI after the PALOMA-2 trial showed an improvement in progression-free survival (PFS) for the experimental arm (palbociclib and letrozole) compared with an AI alone [4]. Subsequently, ribociclib and abemaciclib exhibited remarkably similar outcomes when used in combination with endocrine therapy (ET) [5,6,7,8,9]. CDK4/6 inhibitors are oral medications, well-tolerated, and do not negatively impact quality of life [10,11,12]. 

In this study, we present population-based outcomes for first-line treatment with a CDK4/6 inhibitor combined with an AI amongst patients with HR-positive, HER2-negative MBC in the province of Alberta, Canada. 

## 2. Materials and Methods

### 2.1. Study Design

This was a retrospective, population-based, cohort study of patients with HR-positive, HER2-negative MBC who received a CDK4/6i in combination with an AI as first-line treatment in the metastatic setting. The study protocol was approved by the Health Research Ethics Board of Alberta, Cancer Committee.

### 2.2. Study Population

All patients aged 18 years or older with histologically confirmed HR-positive, HER2-negative advanced or metastatic breast cancer prescribed at least one cycle of a CDK4/6i in combination with an AI in the first-line setting from 1 January 2016 through 30 June 2019 in the province of Alberta, Canada were included. 

### 2.3. Data Sources

Patients were identified from the Alberta Health Services (AHS) Cancer Control Breast Data Mart (BDM). The BDM is a data repository of all breast cancer patients diagnosed from 1 January 2004 onwards in Alberta and includes information on patient demographics, tumour characteristics, surgical intervention, Cancer Control Alberta clinic visits, systemic therapies prescribed, and vital status. The following variables were extracted from the BDM: date of birth, tumour characteristics, vital status, date and cause of death if deceased, and date of last contact with Cancer Control Alberta or Alberta Health if not deceased. Review of the electronic medical record was performed to obtain further disease and treatment variables. The cohort was retrieved from the BDM on 14 April 2020 based on CDK4/6i prescription between 1 January 2016 through 30 June 2019, and chart review occurred through 19 August 2020. 

### 2.4. Outcomes

The primary endpoint was progression-free survival (PFS), defined as the time from treatment initiation with a CDK4/6i until the first documented progression (as per physician notes or most relevant diagnostic imaging report), death from any cause, or last follow-up. Secondary time to event outcomes included PFS2 (time from the start of CDK4/6i until progression on the treatment following CDK4/6i + ET, death from any cause, or last follow-up), time to progression (TTP) on second-line therapy (time from the start of second-line therapy to next progression, death from any cause, or last follow-up), and overall survival (time from the start of CDK4/6i until death from any cause or last follow-up). Additional outcomes of interest included the timing of CDK4/6i initiation relative to the start of an AI, CDK4/6i dose reduction, median time on a CDK4/6i, discontinuation of CDK4/6i therapy due to toxicity, and distribution of second-line therapies. 

### 2.5. Statistical Analysis

Descriptive statistics were used to summarize patient demographics plus tumour and treatment characteristics. Categorical data were described using frequencies and percentages, whereas continuously scaled measures were summarized by the median and interquartile range values. Survival distributions were estimated using the Kaplan–Meier method. A multivariate analysis using the Cox proportional hazards model was constructed to determine the associations between potentially prognostic clinical variables and progression-free survival. We aimed to include potentially prognostic variables applicable to the entire cohort. We included some variables that could potentially identify subgroups with favourable prognosis where CDK4/6i use might be delayed to the second-line setting (i.e., de novo vs. relapsed metastatic breast cancer, non-visceral vs. visceral metastases, PR-positive vs. PR-negative disease). For an age surrogate, we used premenopausal vs. postmenopausal as our real-world adoption of palbociclib in premenopausal patients, extrapolated from PALOMA-2, which only included a postmenopausal population. Finally, we included delayed initiation of CDK4/6i and dose reduction, including upfront dose reduction, as we noted these deviations in real-world practice in comparison with protocols used in the pivotal RCTs. All statistical tests were two-sided, and results were considered statistically significant with a priori conventional definition of *p*-value < 0.05. All data were analysed using STATA statistical software (StataCorp. 2013. Release 13. College Station, TX, USA).

## 3. Results

### 3.1. Patient Characteristics

We identified 316 women who met the inclusion criteria. Patient demographic, disease, and prior treatment characteristics are detailed in Table 1. The median age at metastatic diagnosis was 61 years, and less than one-quarter were premenopausal. Thirty-nine percent of patients had de novo MBC, and amongst those who had relapsed, the median recurrence free interval was 58 months, and two-thirds were not on adjuvant treatment at the time of relapse. Three-fourths presented with 1–2 metastatic sites and around half of the patients had non-visceral disease. 

### 3.2. First-Line CDK4/6 Inhibitor Plus Endocrine Therapy

Palbociclib was administered in 94% of patients, while 5% of patients received ribociclib. The remaining 1% had sequential exposure to both agents. For ET, all patients received an AI, and all premenopausal patients additionally underwent ovarian suppression or oophorectomy. The median time to start CDK4/6i after the metastatic diagnosis was 1.4 months (IQR 0–2.5 months). The median time to CDK4/6i initiation from the start of AI was 0 months (IQR 0–0.9 months). A total of 23 patients (7%) had a delayed CDK4/6i start of more than 90 days from the start of the ET. In this subgroup, the median time to initiation of a CDK4/6i was 7.7 months (IQR: 3.6–15.7 months).

### 3.3. Survival Outcomes

The median follow-up time from advanced diagnosis was 28.1 months. At the time of censoring, 131 patients (42%) had disease progression on a CDK4/6i. Disease progression occurred in the liver (44%), bone (41%), other non-visceral sites (24%), lungs (22%), and central nervous system (5%). The median PFS was 37.9 months (95% CI, 26.7–NR). With 131 events, the median OS was not reached. The 30-month and 36-month OS rates were 74% and 68%, respectively.

### 3.4. Associations of Clinical Variables with Progression-Free Survival

In the Cox model, PR-negative tumour (HR, 2.37; 95% CI, 1.45–3.88; *p* = 0.001) and CDK4/6i dose reduction (HR, 1.51; 95% CI, 1.06–2.16; *p* = 0.022) predicted worse PFS (Table 2). Menopausal status, relapsed disease, visceral metastases, and a gap between the start of CDK4/6i and AI were not significantly associated with PFS.

### 3.5. CDK4/6 Inhibitor Duration, Dose Modification, Discontinuation, and Toxicity

The median time on a CDK4/6i was 19.2 months (IQR 10.3–28.0 months). Dose reduction was observed in 8% of patients upfront and 47% overall. At the time of censoring, a total of 162 patients (51%) had stopped first-line CDK4/6i. The reasons for CDK4/6i discontinuation were disease progression (70%), toxicity (13%), physician recommendation (11%), and patient preference (6%). The most common adverse events leading to treatment discontinuation were nausea (24%), neutropenia (24%), fatigue (19%), increased transaminases (14%), and thrombocytopenia (9.5%). Febrile neutropenia, repeated urinary infections, and pulmonary infection occurred in one patient each, and none of these three patients were hospitalized.

### 3.6. Second-Line Therapy

Of the 131 patients with progression, 117 (89%) received second-line therapy (Table 3). Chemotherapy was the subsequent treatment in 46% of patients, while ET +/− a targeted agent was used in 51%. Median TTP on second-line therapy was 6.3 months (95% CI, 4.0–8.6 months). Median TTP was 9.0 months (95% CI, 5.8–17.6 months) for chemotherapy and 4.0 months (95% CI, 3.4–8.6 months) for ET +/− a targeted agent (*p* = 0.012). In patients who received subsequent therapy, the median PFS2 was 21.8 months (95% CI, 17.8–24.2 months). PSF2 was 20.3 months (95% CI, 16.1–30.3 months) for those who received chemotherapy and 21.8 months (95% CI, 17.8–24.4 months) for those who received ET +/− a targeted agent (*p* = 0.17).

## 4. Discussion

This is the first study to report population-based outcomes for first-line combination CDK4/6i + AI therapy in patients with HR-positive, HER2-negative MBC within a Canadian province. We found a median PFS of 37.9 months (95% CI, 26.7 months–NR), which compares favourably with the pooled median PFS of 28.0 months (95% CI, 25.3–29.1 months) for the CDK4/6i + AI arms of pivotal randomized controlled trials (RCTs) with similar follow-up durations [13]. In our cohort, which was largely exposed to palbociclib, PFS was not associated with menopausal status (postmenopausal vs. premenopausal HR 1.04; 95% CI, 0.67–1.62, *p* = 0.850).

Smaller institution-based retrospective cohorts [14,15,16,17] have estimated median PFS ranging from 15.1 months [14] up to 36.7 months [17] for CDK4/6i + ET. Although the ET backbone in these small studies was largely an AI, a few studies included patients treated with fulvestrant as first-line treatment, suggesting a mix of patients with hormone-resistant disease and less favourable prognoses. The largest retrospective cohort of patients treated purely with first-line CDK4/6i + AI was ascertained from the Flatiron Health Database, which at the time of their analysis had collected information from 2 million cancer patients across 275 cancer centres in the US [18]. Their cohort included 878 patients with a mean age of 65.2 years, and 51% had visceral disease. Median PFS was 21.9 months (95% CI, 20.8–28.2). A prospective German registry study reported the outcomes of 99 patients treated with CDK4/6i + ET and 132 treated with ET alone [19]. Median PFS was 24.7 vs. 16.6 months in favour of combination treatment, but the difference was not statistically significant, with an adjusted hazard ratio (HR) of 0.87 (95% CI, 0.58–1.32), probably reflecting the small sample size of the study. Other prospective real-world studies have now been published, including a multicentre cohort from northern Italy [20]. Of the 182 patients included in the primary analysis, 61 (33.5%) received palbociclib plus an AI or fulvestrant in the first-line setting. As their population was largely pre-treated, the median PFS was 13.0 months. PFS and OS decreased as palbociclib plus ET was used in later lines of treatment. CompLEEment-1 (C-1) was a large, international phase IIIb study that included patients with previously untreated HR-positive, HER2-negative de novo MBC or relapsed disease with at least 12 months duration after neoadjuvant/adjuvant AI exposure. All patients were treated with ribociclib and letrozole and if premenopausal, ovarian function suppression or ablation [21]. Results from the Canadian cohort of 251 women were presented at the European Society of Medical Oncology meeting in 2020. The Canadian C-1 cohort had a median age of 58 years, with 67% having visceral disease. The median time to progression was 26.5 months (95% CI, 22.3–30.1) with efficacy and toxicity data similar in comparison with the global cohort [22].

Median PFS varies across the pivotal RCTs and real-world studies for reasons that may include differences in population/treatment characteristics, frequency and rigorousness of clinical and imaging assessments for progression, and follow-up duration. Several factors may have positively influenced PFS in our cohort. A large proportion of our patients had a single metastatic site (47% compared with 29–34% for pivotal RCTs [4,5,6,7,8,9] and 25% for the C-1 population [22]). While the proportion of patients with visceral metastasis (53%) is in accordance with that of the aforementioned RCTs, 48–59% [4,5,6,7,8,9], this is lower than the proportion reported for C-1 (67%) [22]. Our population may have been subjected to less frequent clinical/imaging assessments for progression in comparison with trial protocols or real-world studies from jurisdictions with privately administered care, and classification of progression was not based strictly on RECIST but rather overall physician evaluation. Finally, our duration of follow-up was relatively long. Use of CDK4/6i + AI therapy for MBC patients with minimal disease burden may reflect access to CDK4/6 inhibitors in Alberta. Expanded access and patient support programs generally included patients who were previously untreated for metastatic disease and had not relapsed at or within 1 year after stopping an adjuvant AI. Subsequently, these criteria were strictly adopted with the onset of public funding. Second-line CDK4/6i with fulvestrant was largely not accessible and only publicly funded at the time of writing this manuscript. Hence, the treating medical oncologists may have tended to prescribe dual therapy to all eligible patients. The optimal sequencing of endocrine therapies plus or minus targeted agents is currently unknown, and with more recent access to CDK4/6 inhibitors beyond the first-line setting, there may be a shift to reserving dual therapy upfront for a potentially worse prognosis population. Further studies (including already established prospective studies such as PRAEGNANT [19], POLARIS [23], and TreatERight [24,25,26]) examining real-world treatment patterns and comparative effectiveness of CDK4/6 inhibitors in the first- vs. second-line setting are warranted.

While median OS in our cohort has not yet been reached, OS at 3 years was 68%. This result is comparable to OS at 3 years for the palbociclib + letrozole cohort from the Flatiron database (72%) [20] and superior to a historical Alberta cohort of de novo HR-positive, HER2-negative MBC patients aged <65 years who were diagnosed 1 January 2010 through 31 December 2014 and would not have had access to a CDK4/6i in the first-line setting (56%) [27]. In terms of the pivotal RCTs of CDK4/6 inhibitors + AI, only Monaleesa-7 (all pre-menopausal patients with ovarian function suppression) reported OS, which was significantly better in the ribociclib arm (OS at 42 months, 70% vs. 46%; HR, 0.71, 95% CI, 0.54–0.95) [28].

As accrual of mortality events across patients with HR-positive, HER2-negative MBC is slow, PFS2 is being considered as a potential surrogate for OS and an outcome to examine whether a new first-line treatment could have a detrimental effect on the duration of response to subsequent therapy [29,30]. PFS2 and related outcomes (e.g., time to third-line treatment) are reported by the pivotal RCTs that continue to demonstrate the superiority of the CDK4/6i + AI arm [28,31,32]. These updated analyses show that the use of a CDK4/6i, when compared with placebo, prolonged PFS2 and time to subsequent therapy. Although more data is needed to validate the importance of PFS2 in relation to OS in HR-positive, HER2-negative metastatic breast cancer, postponing chemotherapy and its inherent toxicity is an important outcome on its own. Further, if upfront use of a CDK4/6i truly does not impair next-line treatment, it can be hypothesized that delaying CDK4/6i use to the second-line setting may be reasonable in a prognostically favourable subset of patients. In our analysis, PFS2 was 21.8 months, lower than the PFS for first-line CDK4/6i (37.9 months). Only 37% (117) patients from our cohort received second line therapy after the first progression, which could explain this result. There was no difference in PFS2 for patients who received ET +/− targeted therapy compared with chemotherapy. Almost half of the patients in our cohort who received a second-line treatment during the follow-up period were prescribed chemotherapy (46%), which is a higher proportion than that reported for post-CDK4/6i + AI progression in the pivotal RCTs (15.8–36.6%) [28,31,32,33]. The use of second-line chemotherapy likely relates to the lack of public funding for fulvestrant and the decision to remove funding for exemestane + everolimus for patients with prior CDK4/6i exposure in Alberta. Such therapies were accessible for limited periods of time through compassionate access programs or private medication insurance plans for a minority of patients. Fortunately, many clinical trials are underway to help establish standard ET +/− targeted therapy post-CDK4/6 + AI [34].

In this study, 92% of patients started a CDK4/6i at full dose and dose reductions (47%), consistent with rates reported in the pivotal RCTs (31–55%) [4,6,9]. We found that dose reduction was significantly associated with worse PFS. This is in contrast to what has been observed in randomized trials [35,36,37,38] and some other retrospective studies [39,40]. Upfront dose reduction and subsequent dose reduction due to toxicity could reflect poor patient-related prognostic factors, such as less favourable performance status and comorbidities. Although we were unable to adjust for these patient-related variables, our CDK4/6i discontinuation rate due to toxicity was slightly higher (13%) than that encountered in the RCTs (4–10%) [4,6,9] and other retrospective studies (2–10%) [14,15,17,22,41]. It follows that if PFS had also been adjusted for performance status and comorbidities, CDK4/6i dose reduction may not have been significantly associated with a worse outcome. Only three patients stopped treatment due to an infectious complication, and, reassuringly, none of them required hospitalization. This could mean that discontinuation of CDK4/6i due to toxicity may be driven by persistent lower grade adverse events rather than single serious adverse events.

Strengths of our real-world study include the use of population-level data, novel investigation of delayed CDK4/6i start as a variable in the survival analyses, and inclusion of subsequent therapy information. This study had several limitations as well. Our population-level data were likely incomplete. The BDM only includes patients diagnosed within the province from 2004 onwards. Hence, patients diagnosed with early-stage disease prior to 2004 who relapsed during our accrual period would not have been captured. Further, patients diagnosed outside Alberta but subsequently treated within the province would also have been missed. As mentioned previously, survival outcomes were not adjusted for ECOG performance status or comorbidities due to difficulty in collecting these variables retrospectively. Omission of these variables in the Cox regression may explain the significant association between CDK4/6i dose reduction and worse PFS. We did not have access to ethnicity information, and given our small population, we did not include variables that related to relapsed patients only, such as recurrence-free interval and exposure to systemic treatments in the adjuvant setting. Omission of potentially prognostic variables in the multivariable analysis could limit the generalizability of our results. Radiologic progression was not defined by RECIST criteria but mostly as per the treating physician, which carries an inherent bias. Finally, we did not have a concurrent cohort of patients treated with AI monotherapy. As previously mentioned, CDK4/6 inhibitors were only available with an AI in the first-line setting, and hence, most eligible patients would have been offered this treatment combination. Patients offered an AI alone during the study era would have likely been older with more comorbidities, yielding a futile comparison.

## 5. Conclusions

The adoption of CDK4/6i + AI as a first-line treatment for HR-positive, HER2-negative MBC in Alberta is justified based on our favourable PFS and early OS outcomes.

## Figures and Tables

**Table 1:** Baseline characteristics of patients.

Characteristic	Number (*n* = 316)	Percent
**Age at metastatic diagnosis, years**		
Median	61	
Interquartile range	53–70	
**Age categories, years**		
≤65	198	62.70%
>65	118	37.30%
**Menopausal status**		
Premenopausal	58	18.40%
Postmenopausal	258	81.70%
**Histology**		
Ductal	225	71.20%
Lobular	39	12.30%
Mixed	28	8.90%
Other	24	7.60%
**Grade (*n* = 228)**		
I	23	10.10%
II	100	43.90%
III	105	46.00%
**Estrogen receptor status**		
Positive	311	98.40%
Unknown	5	1.60%
**Progesterone receptor status**		
Positive	280	88.60%
Negative	31	9.80%
Unknown	5	1.60%
**De novo metastatic**		
Yes	123	38.90%
No	193	61.10%
**Recurrence-free interval (*n* = 193)**		
<1 year	30	15.50%
1–2 years	24	12.40%
>2 years	139	72.00%
**Prior surgery (*n* = 193)**		
Yes	174	90.20%
No	19	9.80%
**Adjuvant radiotherapy (*n* = 193)**		
Yes	105	54.40%
No	88	45.60%
**Adjuvant endocrine therapy (*n* = 193)**		
Yes	121	62.70%
No	72	37.30%
**Relapse on prior therapy (*n* = 193)**		
Relapsed on tamoxifen	52	26.90%
Relapsed on aromatase inhibitor	10	5.20%
Relapsed on chemotherapy	2	1.00%
Not on therapy	127	65.80%
Not known	2	1.00%
**Disease extent**		
Inoperable locally advanced	5	1.60%
Metastatic	311	98.40%
**Number of metastatic sites (*n* = 311)**		
1	146	46.90%
2	98	31.50%
≥3	67	21.40%
**Metastatic distribution (*n* = 311)**		
Visceral	164	52.70%
Non-visceral only	147	47.30%
**Metastatic sites (*n* = 311)**		
Bone	85	26.90%
Other non-visceral	134	42.40%
Lung	117	37.00%
Liver	49	15.50%
Brain/CNS	3	1.00%
Other	28	8.90%

**Table 2:** Multivariable Cox model for PFS.

Variable	HR	95% CI	*p*-Value
**Menopausal status**			
Pre	Ref		
Post	1.04	0.67–1.62	0.85
**De novo metastatic**			
Yes	Ref		
No	1.22	0.85–1.76	0.268
**CDK4/6i within 90 days**			
No	Ref		
Yes	1.14	0.61–2.11	0.686
**Metastases**			
Non-visceral	Ref		
Visceral	1.14	0.80–1.62	0.453
**Dosing**			
No decrease	Ref		
Decrease	1.51	1.06–2.16	0.022
**PR expression**			
Positive	Ref		
Negative	2.37	1.45–3.88	0.001

Abbreviations: CI, confidence interval; HR, hazard ratio; PR, progesterone receptor; Ref, reference.

**Table 3:** Second-line therapies.

Choice of Therapy	Number (*n* = 117)	Percent
Single agent aromatase inhibitor	25	21.4%
Tamoxifen	5	4.3%
Fulvestrant	11	9.4%
Fulvestrant + CDK4/6i	3	2.6%
Endocrine therapy + everolimus	15	12.8%
Capecitabine	36	30.8%
Intravenous chemotherapy	18	15.4%
Other	4	3.4%

Abbreviations: CDK4/6i, cyclin-dependent kinase 4/6 inhibitor.

## Data Availability

The data that support the findings of this study are available on request from the corresponding author. The data are not publicly available due to privacy or ethical restrictions.

## References

[1] World Health Organization International Agency for Research on Cancer The Global Cancer Observatory. https://www.wcrf.org/dietandcancer/cancer-trends/worldwide-cancer-data.

[2] Canadian Cancer Statistics. Breast Cancer Statistics. https://www.cancer.ca/en/cancer-information/cancer-type/breast/statistics/?region=on.

[3] Surveillance, Epidemiology, and End Results (SEER) Program (www.seer.cancer.gov). https://seer.cancer.gov/statfacts/html/breast-subtypes.html.

[4] Finn R.S., Martin M., Rugo H.S., Jones S., Im S.A., Gelmon K., Harbeck N., Lipatov O.N., Walshe J.M., Moulder S. (2016). Palbociclib and Letrozole in Advanced Breast Cancer. N. Engl. J. Med..

[5] Hortobagyi G.N., Stemmer S.M., Burris H.A., Yap Y.S., Sonke G.S., Paluch-Shimon S., Campone M., Blackwell K.L., André F., Winer E.P. (2016). Ribociclib as First-Line Therapy for HR-Positive, Advanced Breast Cancer. N. Engl. J. Med..

[6] Hortobagyi G.N., Stemmer S.M., Burris H.A., Yap Y.S., Sonke G.S., Paluch-Shimon S., Campone M., Petrakova K., Blackwell K.L., Winer E.P. (2018). Updated results from MONALEESA-2, a phase III trial of first-line ribociclib plus letrozole versus placebo plus letrozole in hormone receptor-positive, HER2-negative advanced breast cancer. Ann. Oncol..

[7] Goetz M.P., Toi M., Campone M., Sohn J., Paluch-Shimon S., Huober J., Park I.H., Trédan O., Chen S.C., Manso L. (2017). MONARCH 3: Abemaciclib As Initial Therapy for Advanced Breast Cancer. J. Clin. Oncol..

[8] Johnston S., Martin M., Di Leo A., Im S.A., Awada A., Forrester T., Frenzel M., Hardebeck M.C., Cox J., Barriga S. (2019). MONARCH 3 final PFS: A randomized study of abemaciclib as initial therapy for advanced breast cancer. NPJ Breast Cancer..

[9] Tripathy D., Im S.A., Colleoni M., Franke F., Bardia A., Harbeck N., Hurvitz S.A., Chow L., Sohn J., Seok Lee K. (2018). Ribociclib plus endocrine therapy for premenopausal women with hormone-receptor-positive, advanced breast cancer (MONALEESA-7): A randomised phase 3 trial. Lancet Oncol..

[10] Rugo H.S., Diéras V., Gelmon K.A., Finn R.S., Slamon D.J., Martin M., Neven P., Shparyk Y., Mori A., Lu D.R. (2018). Impact of palbociclib plus letrozole on patient-reported health-related quality of life: Results from the PALOMA-2 trial. Ann. Oncol..

[11] Harbeck N., Franke F., Villanueva-Vazquez R., Lu Y.S., Tripathy D., Chow L., Babu G.K., Im Y.H., Chandiwana D., Gaur A. (2020). Health-related quality of life in premenopausal women with hormone-receptor-positive, HER2-negative advanced breast cancer treated with ribociclib plus endocrine therapy: Results from a phase III randomized clinical trial (MONALEESA-7). Ther. Adv. Med. Oncol..

[12] Fasching P.A., Bardia A., Nusch A., Jerusalem G., Chan A., El Saghir N., Alba E., Im S., Janni W., Chandiwana D. Pooled Analysis of Patient-reported Quality of Life in the MONALEESA-2, -3, and -7 Trials of Ribociclib Plus Endocrine Therapy to Treat Hormone Receptor–positive, HER2-Negative Advanced Breast Cancer. Presented at the European Society for Medical Oncology (ESMO), Virtual Congress.

[13] Gao J.J., Cheng J., Bloomquist E., Sanchez J., Wedam S.B., Singh H., Amiri-Kordestani L., Ibrahim A., Sridhara R., Goldberg K.B. (2020). CDK4/6 inhibitor treatment for patients with hormone receptor-positive, HER2-negative, advanced or metastatic breast cancer: A US Food and Drug Administration pooled analysis. Lancet Oncol..

[14] Varella L., Eziokwu A.S., Jia X., Kruse M., Moore H.C.F., Budd G.T., Abraham J., Montero A.J. (2019). Real-world clinical outcomes and toxicity in metastatic breast cancer patients treated with palbociclib and endocrine therapy. Breast Cancer Res. Treat..

[15] Fountzilas E., Koliou G.A., Vozikis A., Rapti V., Nikolakopoulos A., Boutis A., Christopoulou A., Kontogiorgos I., Karageorgopoulou S., Lalla E. (2020). Real-world clinical outcome and toxicity data and economic aspects in patients with advanced breast cancer treated with cyclin-dependent kinase 4/6 (CDK4/6) inhibitors combined with endocrine therapy: The experience of the Hellenic Cooperative Oncology Group. ESMO Open.

[16] Xi J., Oza A., Thomas S., Ademuyiwa F., Weilbaecher K., Suresh R., Bose R., Cherian M., Hernandez-Aya L., Frith A. (2019). Retrospective Analysis of Treatment Patterns and Effectiveness of Palbociclib and Subsequent Regimens in Metastatic Breast Cancer. J. Natl. Compr. Cancar Netw..

[17] Petracci F., Abuin G.G., Pini A., Chacón M. (2020). RENATA study-Latin American prospective experience: Clinical outcome of patients treated with palbociclib in hormone receptor-positive metastatic breast cancer-real-world use. E Cancer Med. Sci..

[18] Torres M., Liu X., Mardekian J., McRoy L. (2019). Palbociclib Plus an Aromatase Inhibitor as First-Line Therapy for Metastatic Breast Cancer in US Clinical Practice: Real-World Progression-Free Survival Analysis. In:European Society for Medical Oncology (ESMO) Congress, September 29th, 2019, Barcelona, Spain (Abstract # 3536). Ann. Oncol..

[19] Schneeweiss A., Ettl J., Lüftner D., Beckmann M.W., Belleville E., Fasching P.A., Fehm T.N., Geberth M., Häberle L., Hadji P. (2020). Initial experience with CDK4/6 inhibitor-based therapies compared to antihormone monotherapies in routine clinical use in patients with hormone receptor positive, HER2 negative breast cancer—Data from the PRAEGNANT research network for the first 2 years of drug availability in Germany. Breast.

[20] Palumbo R., Torrisi R., Sottotetti F., Presti D., Rita Gambaro A., Collovà E., Ferzi A., Agostinetto E., Maria Teragni C., Saltalamacchia G. (2021). Patterns of treatment and outcome of palbociclib plus endocrine therapy in hormone receptor-positive/HER2 receptor-negative metastatic breast cancer: A real-world multicentre Italian study. Ther. Adv. Med. Oncol..

[21] De Laurentiis M., Jimenez M.M., Ring A., Cottu P., Zhou K., Wu J., Zarate J.P., Zamagni C. (2017). CompLEEment-1: Phase 3b study of ribociclib + letrozole for the treatment of hormone receptor-positive (HR+), human epidermal growth factor receptor 2-negative (HER2–) advanced breast cancer (ABC) in patients with no prior endocrine therapy (ET) for ABC. In: European Society for Medical Oncology (ESMO) Congress, 11 September 11 2017, Madrid, Spain (Abstract # 4272). Ann. Oncol..

[22] Califaretti N., Ferrario C., Warner E., Joy A.A., Chia S.K.L., Wu J., Zarate J.P., Menon-Singh L., Leite R.A., Haftchenary S. (2020). Updated results from the Canadian sub-population of the phase IIIb CompLEEment-1 ribociclib + letrozole HR+ HER2- advanced breast cancer trial. In:European Society for Medical Oncology (ESMO) Virtual Congress, September 17th, 2020, (Abstract # 317P). Ann. Oncol..

[23] Tripathy D., Blum J.L., Rocque G.B., Bardia A., Karuturi M.S., Cappelleri J.C., Liu Y., Zhang Z., Davis K.L., Wang Y. (2020). POLARIS: A prospective, multicenter, noninterventional study assessing palbociclib in hormone receptor-positive advanced breast cancer. Future Oncol..

[24] Ferrario C., Califaretti N., Doyle C., Dent S.F., Roy J.A., Perri S.R., Chia S.K.L. (2017). Treat ER+ight: Canadian prospective observational study in post-menopausal HR+HER2- advanced breast cancer women—First interim analysis (IA). J. Clin. Oncol..

[25] Dent S., Califaretti N., Doyle C., Ferrario C., Chouinard E., Kulkarni S., Roy J.A., Perri S.R., Chia S. Treat ER+ight Canadian prospective observational study in HR+ advanced breast cancer: 2nd interim analysis. Abstract P3-15-02. Proceedings of the 2017 San Antonio Breast Cancer Symposium.

[26] Doyle C., Califaretti N., Dent S., Iqbal N., Mates M., Kulkarni S., Bains P., Glenns V., Perri S.R., Chia S. (2019). Treat ER+ight Canadian prospective observational study in HR+ advanced breast cancer: 3rd interim analysis [abstract]. In: Proceedings of the 2018 San Antonio Breast Cancer Symposium; 2018 Dec 4-8; San Antonio, TX. Philadelphia (PA): AACR. Cancer Res..

[27] Kornaga E.N., Matutino A.R., Pereira A.A., Verma S., Lupichuk S. Survival in women with de novo metastatic breast cancer: Comparison of real-world evidence from a publicly funded Canadian province and the United States by insurance status. Proceedings of the San Antonio Breast Cancer Symposium.

[28] Im S.A., Lu Y.S., Bardia A., Harbeck N., Colleoni M., Franke F., Chow L., Sohn J., Lee K.S., Campos-Gomez S. (2019). Overall Survival with Ribociclib plus Endocrine Therapy in Breast Cancer. N. Engl. J. Med..

[29] Matulonis U.A., Oza A.M., Ho T.W., Ledermann J.A. (2015). Intermediate clinical endpoints: A bridge between progression-free survival and overall survival in ovarian cancer trials. Cancer.

[30] Mainwaring P.N., Zhang L., Mundle S.D., Liu K., Pollozi E., Gray A., Wildgust M. (2019). Correlation of progression free survival-2 and overall survival in solid tumors. In: Proceedings of the the European Society for Medical Oncology (ESMO) Congress, September 28th, 2019, Barcelona, Spain (Abstract # 2935). Ann. Oncol..

[31] Martín M., Johnston S., Huober J., Di Leo A., Sohn J., Andre V.A., Martin H.R., Hardebeck M.C., Goetz M.P. (2019). MONARCH 3: Updated time to chemotherapy and disease progression following abemaciclib plus aromatase inhibitor (AI) in HR+, HER2- advanced breast cancer (ABC). Ann. Oncol..

[32] Rugo H.S., Finn R.S., Diéras V., Ettl J., Lipatov O., Joy A.A., Harbeck N., Castrellon A., Iyer S., Lu D.R. (2019). Palbociclib plus letrozole as first-line therapy in estrogen receptor-positive/human epidermal growth factor receptor 2-negative advanced breast cancer with extended follow-up. Breast Cancer Res. Treat..

[33] Blackwell K.L., Paluch-Shimon S., Campone M., Conte P., Petrakova K., Favret A., Blau S., Beck J.T., Miller M., Sutradhar S. (2018). Subsequent treatment for postmenopausal women with hormone receptor-positive, HER2-negative advanced breast cancer who received ribociclib + letrozole vs placebo + letrozole in the phase III MONALEESA-2 study. In: San Antonio Breast Cancer Symposium, San Antonio TX. December 5-9th 2017. Abstract P5-21-18. Cancer Res..

[34] Xi J., Ma C.X. (2020). Sequencing Endocrine Therapy for Metastatic Breast Cancer: What Do We Do After Disease Progression on a CDK4/6 Inhibitor?. Curr. Oncol. Rep..

[35] Verma S., Bartlett C.H., Schnell P., DeMichele A.M., Loi S., Ro J., Colleoni M., Iwata H., Harbeck N., Cristofanilli M. (2016). Palbociclib in Combination With Fulvestrant in Women with Hormone Receptor-Positive/HER2-Negative Advanced Metastatic Breast Cancer: Detailed Safety Analysis from a Multicenter, Randomized, Placebo-Controlled, Phase III Study (PALOMA-3). Oncologist.

[36] Diéras V., Harbeck N., Joy A.A., Gemon K.A., Ettl J., Verma S., Lu D., Gauthier E.R., Schnell P., Mori A. PALOMA-2: Neutropenia patterns in patients with estrogen receptor−positive/human epidermal growth factor receptor 2−negative first-line advanced breast cancer receiving palbociclib plus letrozole. Proceedings of the 42nd Congress of the European Society for Medical Oncology.

[37] Zheng J., Yu Y., Durairaj C., Diéras V., Finn R.S., Wang D.D. (2021). Impact of Dose Reduction on Efficacy: Implications of Exposure-Response Analysis of Palbociclib. Target Oncol..

[38] Sun W., Yu Y., Hoffman J., Turner N.C., Cristofanilli M., Wang D. Palbociclib exposure-response analyses in second-line treatment of hormone receptor−positive advanced breast cancer. Proceedings of the American Society of Clinical Oncology Annual Meeting.

[39] Clifton K.K., Kimmel J., Yi M., Chad B., Litton J., Debu T., Meghan K. The impact of dose delays and reductions on toxicity and progression free survival (PFS) in patients receiving palbociclib [abstract]. Proceedings of the 2017 San Antonio Breast Cancer Symposium.

[40] Wilkie J., Schickli M.A., Berger M.J., Lustberg M., Reinbolt R., Noonan A., Ramaswamy B., Sardesai S., VanDeusen J., Wesolowski R. (2020). Progression-Free Survival for Real-World Use of Palbociclib in Hormone Receptor-Positive Metastatic Breast Cancer. Clin. Breast Cancer.

[41] Waller J., Mitra D., Mycock K., Taylor-Stokes G., Milligan G., Zhan L., Iyer S. (2019). Real-World Treatment Patterns and Clinical Outcomes in Patients Receiving Palbociclib for Hormone Receptor-Positive, Human Epidermal Growth Factor Receptor 2-Negative Advanced or Metastatic Breast Cancer in Argentina: The IRIS Study. J. Glob. Oncol..

